# Leukotriene inhibitors with dexamethasone show promise in the prevention of death in COVID-19 patients with low oxygen saturations

**DOI:** 10.1017/cts.2022.401

**Published:** 2022-05-16

**Authors:** Peter L. Elkin, Skyler Resendez, Sarah Mullin, Bruce R. Troen, Manoj J. Mammen, Shirley Chang, Gillian Franklin, Wilmon McCray, Steven H. Brown

**Affiliations:** 1 Department of Biomedical Informatics, Jacobs School of Medicine and Biomedical Sciences, University at Buffalo, Buffalo, NY, USA; 2 Department of Veterans Affairs, VA Western New York Healthcare System and WNY, VA Research Service, Buffalo, NY, USA; 3 Department of Medicine, Jacobs School of Medicine and Biomedical Sciences, University at Buffalo, Buffalo, NY, USA; 4 Faculty of Engineering, University of Southern Denmark, Odense, Denmark; 5 Department of Biomedical Informatics, Vanderbilt University Medical Center, Nashville, TN, USA; 6 Department of Veterans Affairs, Veterans Health Administration, Office of Health Informatics, Washington, DC, USA

**Keywords:** Informatics, drug repurposing, EHR, clinical research informatics, translational medicine

## Abstract

**Introduction:**

COVID-19 is a major health threat around the world causing hundreds of millions of infections and millions of deaths. There is a pressing global need for effective therapies. We hypothesized that leukotriene inhibitors (LTIs), that have been shown to lower IL6 and IL8 levels, may have a protective effect in patients with COVID-19.

**Methods:**

In this retrospective controlled cohort study, we compared death rates in COVID-19 patients who were taking a LTI with those who were not taking an LTI. We used the Department of Veterans Affairs (VA) Corporate Data Warehouse (CDW) to create a cohort of COVID-19-positive patients and tracked their use of LTIs between November 1, 2019 and November 11, 2021.

**Results:**

Of the 1,677,595 cohort of patients tested for COVID-19, 189,195 patients tested positive for COVID-19. Forty thousand seven hundred one were admitted. 38,184 had an oxygen requirement and 1214 were taking an LTI. The use of dexamethasone plus a LTI in hospital showed a survival advantage of 13.5% (CI: 0.23%–26.7%; p < 0.01) in patients presenting with a minimal O_2_Sat of 50% or less. For patients with an O_2_Sat of <60 and <50% if they were on LTIs as outpatients, continuing the LTI led to a 14.4% and 22.25 survival advantage if they were continued on the medication as inpatients.

**Conclusions:**

When combined dexamethasone and LTIs provided a mortality benefit in COVID-19 patients presenting with an O_2_ saturations <50%. The LTI cohort had lower markers of inflammation and cytokine storm.

## Introduction

As of January 3, 2022, over 826,000 people in the USA have died from COVID-19 [1] – a rate roughly ten times greater than seasonal influenza deaths [2]. Over 33 million have contracted the infection and a substantial portion of those suffered persisting post-acute symptoms [3]. There is a pressing world-wide need for effective therapies [4,5]. Mostly who die from the COVID-19 suffer from acute respiratory distress syndrome (ARDS) and respiratory insufficiency related to the associated cytokine storm [6,7].

**Fig. 1.**
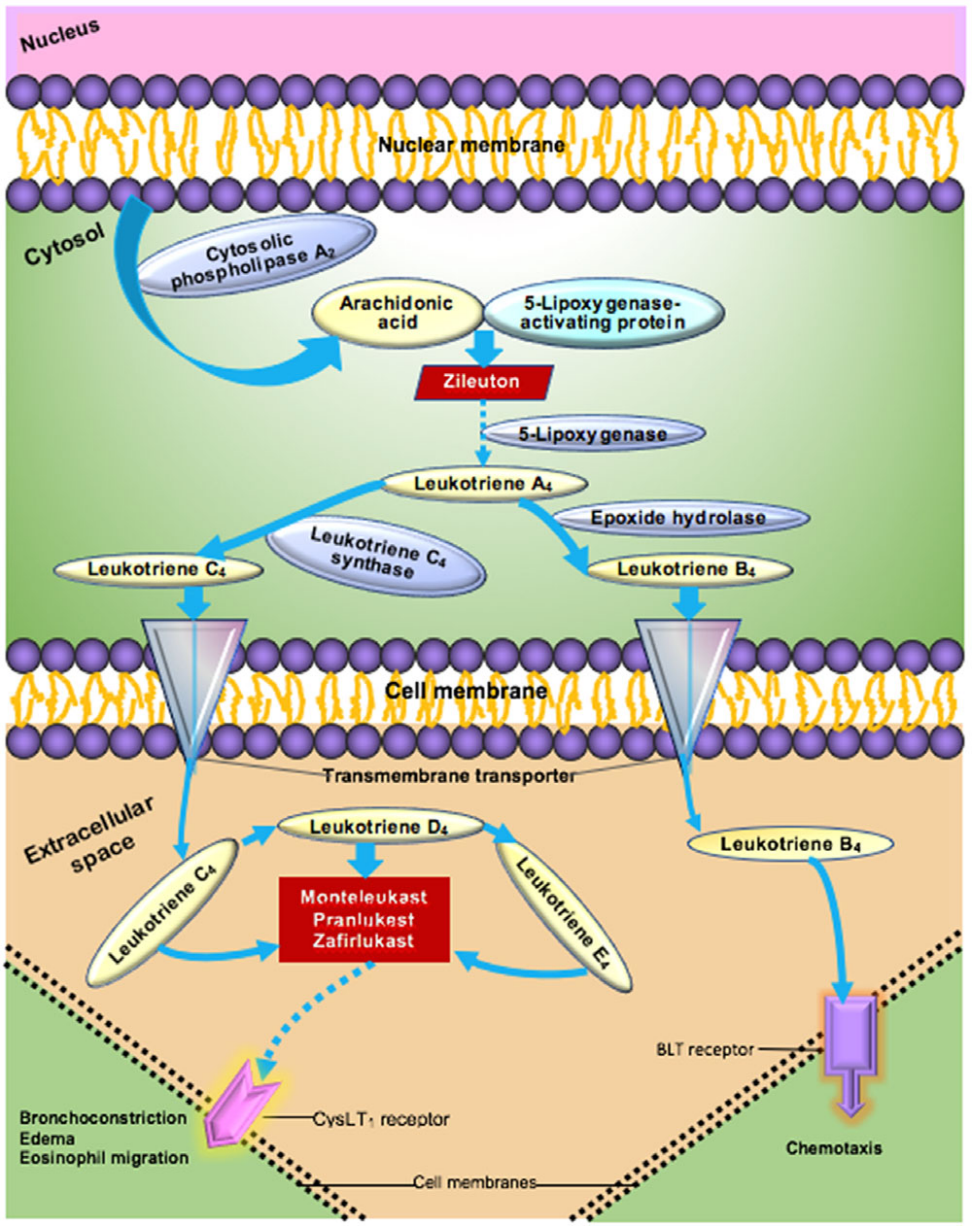
Leukotriene pathway to airway inflammation [10,11].

Cytokines including interleukin 6 (IL6) and interleukin 8 (IL8) have been associated with adverse outcomes including increased death rates in ARDS [8,9]. Leukotrienes modulate a wide range of cytokines via interactions with monocytes, macrophages, eosinophils and other cells in the immune system [10]. The leukotriene pathway begins with arachidonic acid which is transformed to leukotriene A4 by 5-lipoxygenase. Leukotriene A4 is converted to the cysteinyl leukotriene C4 via the action of leukotriene C4 synthase (See Fig. 1) [11]. Cysteinyl leukotrienes (C4, D4, and E4) mediate most of the clinically significant effects of leukotrienes through binding to the cysteinyl leukotriene receptor type 1 (CysLT1), which causes the contraction of human airway smooth muscle, chemotaxis, and increased vascular permeability. Montelukast, pranlukast, and zafirukast are leukotriene inhibitors (LTIs) that interfere with the binding of cysteinyl leukotrienes to the CysLT1 receptor.

It has been known for years that CysLT1 antagonists such as montelukast modulate the production of pro-inflammatory mediators (See Fig. 1) [12,13]. Labeled indications for these medications include prophylaxis and chronic treatment of asthma, prevention of exercise-induced bronchospasm and symptomatic relief of allergic rhinitis. Recent studies have found beneficial effects of CysLT1 antagonists on injury of the blood–brain barrier [14], endothelial dysfunction in tissue culture [15], bronchopulmonary dysplasia induced by hyperoxia [16], and in acute lung inflammation in a mouse model [17]. CysLT1 antagonists have been shown to lower IL6 and IL8 levels in upper respiratory infections and related models [17–19], markers of poor prognosis in ARDS.

Manuscripts published after the start of our study have posited a potential therapeutic role of montelukast in COVID-19 [19–21]. In a review article entitled “Tackling the cytokine storm in COVID-19, challenges and hopes” Abdin et al. note in “It’s worth mentioning that montelukast might have antiviral activity, as it targets SARS-CoV-2 3CL protease and fits properly with stable conformation [22]. Indeed, montelukast exhibited antiviral activity against a wide range of viruses, including Zika virus (ZIKV), DENV-2, and yellow fever virus (YFV) [23]. The use of montelukast, a potent well-known leukotriene modifier, in SARS-CoV-2 may control and prevent the stage of cytokine storm with potential antiviral activity as well” [6]. A small retrospective study by Khan et al. found that COVID-19 patients receiving an LTI had fewer events of clinical deterioration than patients who were not treated with an LTI [24].

Asthma is one of the most common indications for montelukast. There is emerging evidence that asthma itself may be protective in cases of COVID-19. Ciprandi notes a paucity of pediatric patients with asthma and medication-controlled eosinophilia among COVID-19 patients in Italy [25]. Ferastraoaru et al. found that patients with asthma and pre-existing eosinophilia (absolute eosinophil count (AEC) >150 cells/microliter) were less likely to die than asthmatics with AEC <150. They also found that asthmatics without eosinophilia had mortality similar to non-asthmatic patients [26]. Despite these potential confounding factors, we hypothesized that LTIs may have a protective effect in COVID-19 patients.

## Methods

We conducted a retrospective controlled cohort study of 37,495 US veterans with COVID-19 and supplemental oxygen requirements to compare death rates in COVID-19 patients taking LTIs along with dexamethasone with those who were not taking LTIs.

We used data from the Department of Veterans Affairs (VA) Corporate Data Warehouse (CDW) with a focus on a subset called the National Surveillance Tool (NST) [27,28]. The VA CDW is an enterprise resource that contains over 60 clinical domains that are updated nightly from all VA VistA (Veterans Information System Technology Architecture) systems and numerous other clinical and administrative sources in a relational database [29]. CDW is used for a variety of purposes in VA including business management, clinical and administrative research and quality improvement.

The NST is a data mart within the CDW created during the COVID-19 pandemic to be the single authoritative VA data source for outbreaks. It harmonizes data from a variety of sources for patient information, system capacity, staffing, and inventory. Its clinical data sources include VistA inpatient Admission Discharge Transfer (ADT) records, outpatient visits, clinicians’ notes, orders, labs, and medications. The NST was designed to help guide strategic, operational, and tactical response to an outbreak and is a cooperative effort of numerous VA Offices.

**Fig. 2.**
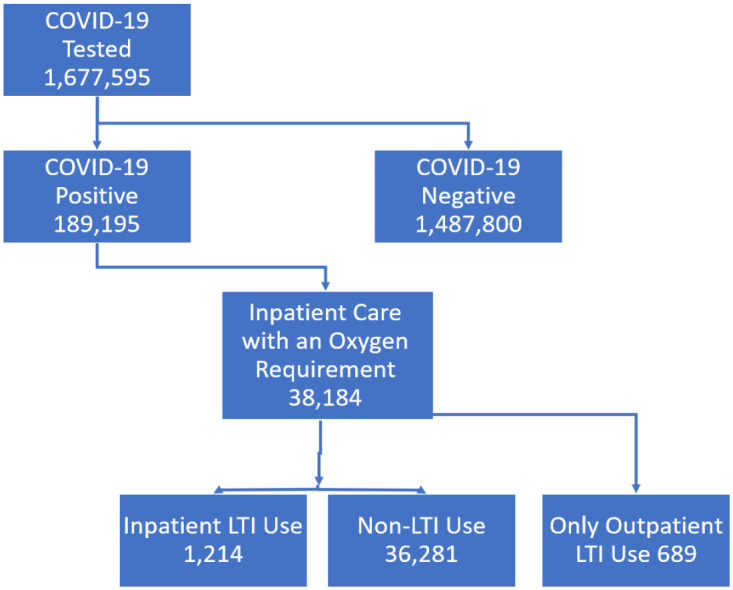
Data flow diagram for the study. LTI: leukotriene inhibitors.

We accessed VA COVID-19 data through the VA Health Services Research (VINCI) mirror of the production COVID-19 Data Mart [30]. We extracted 1,677,595 patients who were tested for COVID-19 by Real Time – Polymerase Chain Reaction (RT-PCR) between January 1, 2020 and November 11, 2021. Of these, 189,195 patients tested positive for COVID-19. Of the positive cases, 40,701 were admitted to the hospital and 38,184 patients when admitted had an oxygen requirement. Of these patients, 1214 were taking a LTI (See Fig. 2). We compared death rates for the COVID-19 positive cohort admitted with a Minimal O_2_Sat <50 that were taking LTIs with the COVID-19-positive cohort admitted with a Minimal O_2_Sat <50 that was not taking LTI .

We defined LTI use as having been dispensed medications for at least 70% of days during the prescribing period. Patients taking between 1 day and 69% of the days were excluded from the study. The start of the prescribing period was defined as the latest of either the initial prescription date or November 1, 2019. This date was selected because the emergence of COVID-19 in the USA in February 2020 and the VA practice of issuing 90-day medication supplies. The end of the prescribing period was defined as the earliest of either 2 weeks after their COVID-19 diagnosis date, their COVID-19 death date, or November 1, 2021.

In addition to LTI use, we obtained the following CDW data elements for individual patients: COVID-19 testing date and result, age, sex, race, maximum D-dimer laboratory result after hospitalization, maximum ferritin result after hospitalization, maximum IL6 result after hospitalization, ADT dates and times, diagnoses, and mortality. Since COVID-19 severity and mortality correlate with the degree of underlying comorbidities, we calculated the 2-year Elixhauser comorbidity index using ICD-10-CM diagnosis codes between November 11, 2019 and November 11, 2021 for risk adjustment [31,32].

We controlled for asthma as a potential confounder by performing subgroup analysis comparing in-hospital death rates between asthmatic LTI users and asthmatic non-LTI users. We also compared asthmatic non-LTI user with non-asthmatic non-LTI users. We determined asthma status by the presence of any ICD-10-CM code for asthma in the format J45.XXX within 2 years of November 11, 2021

### Statistical Approach

All statistical analyses were done using R & R-Studio statistical computing software version 3.6 (https://www.r-project.org/). We calculated univariate frequencies to assess the association of LTI use as compared to non-LTI use with baseline patient characteristics and characteristics such as laboratory values, after admission. Pearson Chi-square tests of association were used to determine the association between categorical variables. To compare quantitative features, such as laboratory values, Chi-Square-tests were used to compare means and the Wilcoxon Rank Sum test was used to compare medians when normality could not be assumed. We estimated the effect of LTI treatment on the rate of mortality using the Pearson Chi-Square test. Multiple regression analysis was used to determine significance at the p < 0.05 level, controlling for age, race, gender, 2yr Elixhauser score, asthma diagnosis, vaccination status (Y, N) and dexamethasone and LTIs inpatient, dexamethasone inpatient, LTIs inpatient, and outpatient.

We looked specifically at patients who presented with an O_2_Sat of <60 and at a cut off of <50.

## Results

Out of 1,677,595 patients tested for COVID-19, 38,184 were positive and were admitted to the hospital with an oxygen requirement. Of those, 1214 used a LTI and 36,281 had no leukotriene usage.

The average age of the patients in the LTI cohort was older than those in the non-LTI cohort (See Table 1). The Elixhauser score was significantly higher in the LTI arm of the study. There was a trend toward more Black individuals in the LTI cohort (See Table 1).

**Table 1.** Baseline characteristics of the Leukotriene Inhbitor (LTI) using and non-LTI using cohorts

	Inpatient LTI	Non-LTI
Patients (% or means with 0.95 conf. intervals)	1214	36,281
Age	67.29 (66.58–68.01)	67.73 (67.59–67.88)
Gender		
Males	86.24%	94.34%
Females	13.76%	5.66%
Race		
American Indian/Alaska Native	1.07%	0.90%
Asian	0.91%	0.82%
Black /African American	23.89%	28.49%
Native Hawaiian /Pacific Islander	1.57%	0.96%
Unknown	7.33%	7.08%
White	65.24%	61.76%
Comorbidities (%)		
Asthma	42.26%	5.52%
COPD	53.38%	24.60%
CHF	20.59%	17.49%
CAD	34.35%	30.83%
Diabetes	50.82%	47.44%
CVD	56.01%	50.99%
Cancer	25.45%	26.83%
CKD	26.94%	26.38%
Elixhauser 2 year	13.08 (12.36–13.79)	11.85 (11.72–11.99)
MinOxVitalResult	87.58 (86.52–88.63)	89.73 (89.55–89.90)

Inflammatory markers, mean of maximal value	Inpatient LTI	Non-LTI
D-Dimer (mg/dL)	513.54 (210.49–816.58)	581.49 (517.62–645.35)
Ferritin (ng/dL)	553.79 (496.58–611.00)	743.84 (722.48–765.20)
IL6 (pg/mL)	160.62 (−84.94 to 406.19)	535.96 (−96.45 to 1168.38)

Abbreviated comorbidities include chronic obstructive lung disease (COPD), congestive heart failure (CHF), coronary artery disease (CAD), cardiovascular disease (CVD), and chronic kidney disease (CKD), IL6 (Interleukin 6), MinOxVitalResult (minimum Oxygen Saturation Vital Sign Result).

When we looked at patients who had an O_2_Sat minimum of <60 cut-off, we had 1652 patients who were treated with dexamethasone, 97 of them also took LTIs, 80 that came in on an LTI and were continued on one during hospitalization. Adjusting for age, gender, race, Elixhauser scores and whether they had asthma, we found that the LTIs when taken both before and during hospitalization provided on average a 13.5% (CI: 0.23%–26.7%) protection against death during their hospitalization (p < 0.05).

When we looked at patients who had an O_2_Sat Minimum of <50, we had 1058 patients who were treated with dexamethasone, 67 of them also took LTIs, 57 that came in on an LTI and were continued on one during hospitalization. Adjusting for age, gender, race, Elixhauser scores and whether they had asthma, we found that the LTIs when taken both before and during hospitalization provided on average a 22.2% (CI: 7.0%–37%) protection against death during their hospitalization (p < 0.01). The use of dexamethasone plus a LTI in hospital showed a survival advantage of 13.5% (CI: 0.23%–26.7%; p < 0.01).

The use of dexamethasone without LTIs was not associated with a survival advantage in people presenting with an O_2_Sat of <50% or an O_2_Sat of <60%.

For patients with an O2Sat of <60 and <50% if they were on LTIs as outpatients, continuing the LTI led to a 14.4% and 22.25% survival advantage if they were continued on the medication as inpatients.

The vaccination rate of having received at least one vaccination prior to admission in the LTI cohort was 9.0% and in the non-LTI cohort was 9.3%.

The average of the maximum D-Dimer (513 *vs*. 581 mg/dl), ferritin (554 *vs* 744 ng/dl) and IL6 (61 *vs* 536 pg/ml) values were all lower in the LTI taking group (Table 1). The IL6 levels in the non-LTI using group were significantly more frequently elevated to >40 pg/ml (55% vs 76%, p-value < 0.001; Pearson Chi-Square).

The inpatient death rate for asthmatic LTI users was lower than inpatient death rates for asthmatic non-LTI users (odds ratio 0.517; p-value < 0.001). Asthmatics without LTI use had a significantly higher overall inpatient mortality than the non-asthmatic population without LTI use (odds ratio 8.77; p < 0.001) (See Table 2).

**Table 2.** Asthma subgroup analysis

	Alive at discharge	Inpatient death	Inpatient death rate
Asthma LTI	117	10	0.07874
Asthma non LTI	651	117	0.15234
Non-asthma non LTI	7463	1016	0.11983

LTI: leukotriene Inhibitors.

## Discussion

COVID-19 patients taking LTIs had lower inpatient death rates and lower 28-day inpatient and outpatient death rates than COVID-19 patients not taking LTIs in this large retrospective cohort-controlled study. In patients taking both dexamethasone and LTIs just as inpatients, there was a survival advantage during hospitalization of 13.6% in patients with a minimum O_2_Sat of 50%. LTI users prior to hospitalization should continue them when admitted as they also have a survival advantage. As the O_2_Sat dropped the utility of the addition of an LTI during hospitalization became more significant both for dexamethasone users and for those who took LTIs as an outpatient.

LTI users had significantly higher rates of important comorbidities including chronic obstructive pulmonary disease, coronary artery disease, and diabetes. Advancing age, racial, and ethnic minority group membership are variables that have been linked to worse COVID-19 outcomes [33,34]. The LTI user cohort was on average 4 years older than the non-LTI user cohort and African Americans were represented at a higher rate. There were more ICU patients in the LTI using cohort. Despite these potential disadvantages, LTI users had significantly lower mortality than non-LTI users.

We accounted for the potential confound of a possible protective role for asthma in COVID-19 mortality in two ways. We found that asthmatics had higher death rates than non-asthmatics among COVID-19 patients who were not taking an LTI. We also found that LTI using asthmatics had a reduced risk of inpatient death when compared to non-LTI using asthmatics. These findings support the hypothesis of a protective effect with LTI use and against a protective effect of asthma. There was no further evaluation of our asthmatic patients with absolute eosinophil counts due to lack of data.

We did not perform separate analyses for the use of inhaled steroids. While these medications have been shown to have some impact on the course of COVID-19 [35], we felt that separating the effect of inhaled steroids from the much stronger impact of routinely administered intravenous dexamethasone per Infectious Disease Society of America treatment guidelines would be difficult [36,37].

Our evaluation of potential pathophysiological mechanisms and markers found that the maximum ferritin, D-Dimer, and IL6 levels were lower in the LTI cohort. In a recent article, Magro found that an IL6 of >40 pg/ml is indicative of cytokine storm in COVID-19 patients [38]. The non-LTI taking patients in our study were more likely to have an IL6 level >40 pg/ml. CysLT1 antagonists have been shown to reduce IL-6 and IL-8 levels, and elevations of these markers are poor prognostic indicators in ARDS. Based on these reported observations and our results, CysLT1 antagonists appear to be a plausible treatment for ARDS and cytokine storm in COVID-19 patients.

We used big data from the Department of Veterans Affairs to uncover a potential COVID-19 treatments that could lead to improved clinical outcomes. Furthermore, because monteleukast is an FDA approved, widely available medication with generic equivalent formulations and easily achieved storage requirements [39], the burden of implementation could be minimal. Our approach is not unique. Increasingly, secondary use of electronic health record (EHR) data is seen as a way to obtain answers to research question when randomized prospective double-blind placebo-controlled trials are impractical or when rapid results are needed [40–42].

Patients who presented with COVID and low oxygen saturations and were taking LTIs as outpatients showed as much as a 22.2% survival advantage if their LTI was continued during their inpatient stay.

Confirmation of our findings in retrospective data awaits a randomized prospective controlled trial of LTI in the treatment of COVID-19 patients. We also suggest that research is needed in high-risk groups, such as the elderly and immunosuppressed populations to determine if prophylaxis with LTIs can decrease rates of severe illness and death during the pandemic.

## Conclusions

LTIs when added to dexamethasone provides a 13.5% survival advantage in patients who present with a minimum O_2_Sat of <50%. LTI use prehospitalization and during hospitalization was associated with significant decreases in-hospital mortality in COVID-19 patients with low oxygen saturations of <60%. The LTI using cohort had lower markers of inflammation and cytokine storm. We postulate that reduced inflammation may be the mechanism by which LTIs improve death rate in COVID-19 patients. These retrospective findings need to be confirmed in a prospective randomized placebo-controlled trial. If confirmed, these findings could be of immediate benefit to patients globally, as LTIs include generic drugs that are inexpensive and available in many countries.

## References

[1] Johns Hopkins Coronavirus Resource Center. *COVID-19 Map*. (https://coronavirus.jhu.edu/map.html)

[2] CDC. *Past Seasons Estimated Influenza Disease Burden*. (https://www.cdc.gov/flu/about/burden/past-seasons.html)

[3] Amenta EM , Spallone A , Rodriguez-Barradas MC , El Sahly HM , Atmar RL , Kulkarni PA. Postacute COVID-19: an overview and approach to classification. Open Forum Infectious Diseases 2020; 7 (12): ofaa509. DOI 10.1093/ofid/ofaa509. 33403218 PMC7665635

[4] Kouznetsov VV. COVID-19 treatment: much research and testing, but far, few magic bullets against SARS-CoV-2 coronavirus. European Journal of Medicinal Chemistry 2020; 203: 112647. DOI 10.1016/j.ejmech.2020.112647. 32693298 PMC7362854

[5] HHS News Division. Biden Administration to Invest $3 Billion from American Rescue Plan as Part of COVID-19 Antiviral Development Strategy. HHS.gov, 2021. (https://www.hhs.gov/about/news/2021/06/17/biden-administration-invest-3-billion-american-rescue-plan-as-part-covid-19-antiviral-development-strategy.html)

[6] Abdin SM , Elgendy SM , Alyammahi SK , Alhamad DW , Omar HA. Tackling the cytokine storm in COVID-19, challenges and hopes. Life Sciences 2020; 257 (4): 118054. DOI 10.1016/j.lfs.2020.118054. 32663575 PMC7832727

[7] Fajgenbaum DC , June CH. Cytokine storm. The New England Journal of Medicine 2020; 383 (23): 2255–2273. DOI 10.1056/NEJMra2026131. 33264547 PMC7727315

[8] Butt Y , Kurdowska A , Allen TC. Acute lung injury: a clinical and molecular review. Archives of Pathology & Laboratory Medicine 2016; 140 (4): 345–350. DOI 10.5858/arpa.2015-0519-RA. 27028393

[9] Cameron MJ , Bermejo-Martin JF , Danesh A , Muller MP , Kelvin DJ. Human immunopathogenesis of severe acute respiratory syndrome (SARS). Virus Research 2008; 133 (1): 13–19. DOI 10.1016/j.virusres.2007.02.014. 17374415 PMC7114310

[10] Rola-Pleszczynski M , Stankova J. Cytokine-leukotriene receptor interactions. Scientific World Journal 2007; 7: 1348–1358. DOI 10.1100/tsw.2007.183. 17767354 PMC5901110

[11] Drazen JM , Israel E , O’Byrne PM. Treatment of asthma with drugs modifying the leukotriene pathway. New England Journal of Medicine 1999; 340 (3): 197–206. DOI 10.1056/NEJM199901213400306. 9895400

[12] Maeba S , Ichiyama T , Ueno Y , Makata H , Matsubara T , Furukawa S. Effect of montelukast on nuclear factor kappaB activation and proinflammatory molecules. Annals of Allergy, Asthma & Immunology 2005; 94 (6): 670–674. DOI 10.1016/S1081-1206(10)61326-9. 15984600

[13] Sener G , Sehirli O , Velioğlu-Oğünç A , et al. Montelukast protects against renal ischemia/reperfusion injury in rats. Pharmacological Research 2006; 54 (1): 65–71. DOI 10.1016/j.phrs.2006.02.007. 16584888

[14] Zhou L , Sun X , Shi Y , Liu J , Luan G , Yang Y. Cysteinyl leukotriene receptor type 1 antagonist montelukast protects against injury of blood-brain barrier. Inflammopharmacology 2019; 27 (5): 933–940. DOI 10.1007/s10787-019-00611-7. 31313075

[15] Zhou X , Cai J , Liu W , Wu X , Gao C. Cysteinyl leukotriene receptor type 1 (CysLT1R) antagonist zafirlukast protects against TNF-α-induced endothelial inflammation. Biomedicine & Pharmacotherapy 2019; 111 (2): 452–459. DOI 10.1016/j.biopha.2018.12.064. 30594784

[16] Chen X , Zhang X , Pan J. Effect of montelukast on bronchopulmonary dysplasia (BPD) and related mechanisms. Medical Science Monitor 2019; 25: 1886–1893. DOI 10.12659/MSM.912774. 30862773 PMC6427930

[17] Davino-Chiovatto JE , Oliveira-Junior MC , MacKenzie B , et al. Montelukast, leukotriene inhibitor, reduces LPS-induced acute lung inflammation and human neutrophil activation. Archivos de Bronconeumología 2019; 55 (11): 573–580. DOI 10.1016/j.arbres.2019.05.003. 31257011

[18] Schad C , Gentile DA , Patel A , Koehrsen JM , Schaffner T , Skoner DP. Effect of montelukast on pro-inflammatory cytokine production during naturally acquired viral upper respiratory infections (vURIs) in adults. Journal of Allergy and Clinical Immunology 2008; 121 (2, Supplement 1): S74. DOI 10.1016/j.jaci.2007.12.294.

[19] Mullol J , Callejas FB , Méndez-Arancibia E , et al. Montelukast reduces eosinophilic inflammation by inhibiting both epithelial cell cytokine secretion (GM-CSF, IL-6, IL-8) and eosinophil survival. Journal of Biological Regulators and Homeostatic Agents 2010; 24 (4): 403–411. 21122279

[20] Almerie MQ , Kerrigan DD. The association between obesity and poor outcome after COVID-19 indicates a potential therapeutic role for montelukast. Medical Hypotheses 2020; 143 (6): 109883. DOI 10.1016/j.mehy.2020.109883. 32492562 PMC7255216

[21] Citron F , Perelli L , Deem AK , Genovese G , Viale A. Leukotrienes, a potential target for Covid-19. Prostaglandins, Leukotrienes and Essential Fatty Acids 2020; 161: 102174. DOI 10.1016/j.plefa.2020.102174. 32977289 PMC7495247

[22] Wu C , Liu Y , Yang Y , et al. Analysis of therapeutic targets for SARS-CoV-2 and discovery of potential drugs by computational methods. Acta Pharmaceutica Sinica B 2020; 10 (5): 766–788. DOI 10.1016/j.apsb.2020.02.008. 32292689 PMC7102550

[23] Chen Y , Li Y , Wang X , Zou P. Montelukast, an anti-asthmatic drug, inhibits zika virus infection by disrupting viral integrity. Frontiers in Microbiology 2019; 10: 3079. DOI 10.3389/fmicb.2019.03079. 32082265 PMC7002393

[24] Khan AR , Misdary C , Yegya-Raman N , et al. Montelukast in hospitalized patients diagnosed with COVID-19. Journal of Asthma 2021; 59 (4): 780–786. DOI 10.1080/02770903.2021.1881967. PMC7938648 33577360

[25] Ciprandi G , Licari A , Filippelli G , Tosca MA , Marseglia GL. Children and adolescents with allergy and/or asthma seem to be protected from coronavirus disease 2019. Annals of Allergy, Asthma & Immunology 2020; 125 (3): 361–362. DOI 10.1016/j.anai.2020.06.001. PMC7447212 32859351

[26] Ferastraoaru D , Hudes G , Jerschow E , et al. Eosinophilia in asthma patients is protective against severe COVID-19 illness. The Journal of Allergy and Clinical Immunology: In Practice 2021; 9 (3): 1152–1162.e3. DOI 10.1016/j.jaip.2020.12.045. 33495097 PMC7826039

[27] Soudin M. Overview of VA Data, Information Systems, National Databases & Research Uses, 2017. (https://www.hsrd.research.va.gov/for_researchers/cyber_seminars/archives/2376-notes.pdf)

[28] Stratford D. National Surveillance Tool assesses readiness across VA’s health system. VAntage Point, 2020. (https://www.blogs.va.gov/VAntage/74896/national-surveillance-tool-assesses-readiness-across-vas-health-system/)

[29] Brown SH , Lincoln MJ , Groen PJ , Kolodner RM. VistA—U.S. Department of Veterans Affairs national-scale HIS. International Journal of Medical Informatics 2003; 69 (2-3): 135–156. DOI 10.1016/s1386-5056(02)00131-4. 12810119

[30] VA Informatics and Computing Infrastructure (VINCI). (https://www.hsrd.research.va.gov/for_researchers/vinci/)

[31] Cai M , Liu E , Zhang R , et al. Comparing the performance of Charlson and Elixhauser comorbidity indices to predict in-hospital mortality among a Chinese population. Clinical Epidemiology 2020;12: 307–316. DOI 10.2147/CLEP.S241610. 32256119 PMC7090198

[32] Elixhauser Comorbidity Software, Version 3.7. (https://www.hcup-us.ahrq.gov/toolssoftware/comorbidity/comorbidity.jsp)

[33] Fauci AS , Lane HC , Redfield RR. Covid-19 - navigating the uncharted. New England Journal of Medicine 2020; 382 (13): 1268–1269. DOI 10.1056/NEJMe2002387. 32109011 PMC7121221

[34] CDC. COVID-19 Health Equity Considerations & Racial & Ethnic Minority Groups. Centers for Disease Control and Prevention, 2020. (https://www.cdc.gov/coronavirus/2019-ncov/community/health-equity/race-ethnicity.html)

[35] Armentia A , Cortés SF , Simón AM , et al. Inhaled corticosteroids may have a protective effect against coronavirus infection. Allergologia et Immunopathologia 2021; 49 (1): 113–117. DOI 10.15586/aei.v49i1.40. 33528938

[36] Bhimraj A , Morgan RL , Shumaker AH , et al. COVID-19 Guideline, Part 1: Treatment and Management. (https://www.idsociety.org/practice-guideline/covid-19-guideline-treatment-and-management/)

[37] RECOVERY Collaborative Group, Horby P , Lim WS , et al. Dexamethasone in hospitalized patients with Covid-19. New England Journal of Medicine 2021; 384 (8): 693–704. DOI 10.1056/NEJMoa2021436. 32678530 PMC7383595

[38] Magro G. SARS-CoV-2 and COVID-19: is interleukin-6 (IL-6) the, culprit lesion, of ARDS onset? What is there besides Tocilizumab? SGP130Fc. Cytokine: X 2020; 2 (2): 100029. DOI 10.1016/j.cytox.2020.100029. 32421092 PMC7224649

[39] DailyMed. SINGULAIR- montelukast sodium granule SINGULAIR- montelukast sodium tablet, chewable SINGULAIR- montelukast sodium tablet, film coated. (https://dailymed.nlm.nih.gov/dailymed/drugInfo.cfm?setid=8c166755-7711-4df9-d689-8836a1a70885)

[40] Elkin PL , Trusko BE , Koppel R , et al. Secondary use of clinical data. In: EFMI-STC, 2010, pp. 14–29. 20543306

[41] Mullin S , Anand E , Sinha S , Song B , Zhao J , Elkin PL. Secondary use of EHR: interpreting clinician inter-rater reliability through qualitative assessment. Studies in Health Technology and Informatics 2017; 241: 165–172. 28809201 PMC5698262

[42] *National COVID Cohort Collaborative (N3C)*. National Center for Advancing Translational Sciences, 2020. (https://ncats.nih.gov/n3c)

